# Pseudopollen in *Camellia oleifera* and its implications for pollination ecology and taxonomy

**DOI:** 10.3389/fpls.2022.1032187

**Published:** 2022-11-17

**Authors:** Bin Yuan, Jing-Kun Yuan, Cheng-Gong Huang, Jia-Rui Lian, Yi-Huan Li, Xiao-Ming Fan, De-Yi Yuan

**Affiliations:** ^1^ Key Laboratory of Cultivation and Protection for Non-Wood Forest Trees, Ministry of Education, Central South University of Forestry and Technology, Changsha, China; ^2^ Key Laboratory of Non-Wood Forest Products of State Forestry Administration, Central South University of Forestry and Technology, Changsha, China

**Keywords:** pseudopollen, protein, polysaccharides, *Camellia oleifera* (Theaceae), taxonomic feature

## Abstract

**Background and aims:**

In 1997, Tsou described the special differentiation of the connective tissues of some species of *Theaceae* to produce single-celled powders with unique patterns called pseudopollen. The purpose of this study was to investigate the morphological structure of the pseudopollen of *Camellia oleifera* (Theaceae) and to study the morphology of pseudopollen in seven other *Camellia* species.

**Methods:**

Scanning electron microscopy, paraffin section, light microscopy, transmission electron microscopy, histochemistry.

**Key result:**

*C. oleifera* pseudopollen was similar to normal pollen in macroscopic morphology but different microscopically. The normal pollen was starch-rich and yellow, with mostly reticulate exine ornamentation. In contrast, the pseudopollen was a white powder, single-celled and rich in protein, with parallel unbranched ridge lines on the outer wall, and originated from the parenchyma of the connective tissues. There are also differences in the micro-characteristics of normal and pseudopollen among different species in *Camellia*.

**Conclusion:**

There are great differences in morphological structure between *C. oleifera* and other species in *Camellia* normal pollen and pseudopollen; these results may indicate that the pseudopollen can be used as a taxonomic basis for *Camellia*, and the macroscopic similarity between pseudopollen and pollen and histochemical characteristics of pseudopollen can be a pollination strategy.

## 1 Introduction

Pseudopollen has been reported in Orchidaceae (Joanna et al., 2020), Scrophulinaceae (Hufford, 1995), and Theaceae (Tsou, 1996) as a material mimicking pollinia or the whole anther. The existence of pseudopollen, which is presumed to attract insects, can improve the fruit setting rate to a certain extent and increase the yield (Zheng et al., 2021). Tsou (1997) observed the presence of pseudopollen on honeybees and concluded that the pseudopollen grains satisfy the insect’s great demand for food in the form of a deceptive signal. (Davies et al., 2013) described pseudopollen as a deceptive signal to simulate pollen, with similar morphological characteristics to normal pollen. In addition, Davies et al. (2002) proposed that pseudopollen is a pollination strategy with both reward and deception functions. These observations indicate that pseudopollen may have an essential status in pollination strategies. It has an important research value for species that use seeds as a source of production. Pseudopollen also exists in *C. oleifera* (Hu and Gao, 2022; Yuan et al., 2022a; Yuan et al., 2022b). *C. oleifera* is a small evergreen tree species and produces seeds with edible oils rich in unsaturated fatty acids and vitamin E (Xiong et al., 2019). It is extensively cultivated, but the production of *C. oleifera* is still in short supply (Qin et al., 2018). *C. oleifera* is a typical entomophilous plant with self-incompatibility, and insects play a crucial role in its reproductive success (Liao et al., 2014; Wang et al., 2015). Therefore, determining the pollination strategy of this species is key to increasing its yield. However, the pseudopollen of *C. oleifera* has not been well studied in terms of their role in the pollination strategy.

Theaceae species are abundant, have an Amphi-Pacific disjunct distribution and a high ornamental and economic value (Yan et al., 2021). The pseudopollen in Theaceae appears as a single-celled mealy material with a unique ornamented outer wall formed from parenchyma cells of the connective tissue found in *Franklinia, Camellia*, *Schima*, and *Gordonia* (Tsou, 1997; Iqbal and Wijesekara, 2002). Lyu et al. (2019) reported that, including the tapetal cell layers, cells of the interlocular septum and stomium were blocked in programmed cell death (PCD) associated with anther dehiscence at 15°C. Furthermore, the expressions of genes correlated with PCD of tapetum and stomium were significantly inhibited at 15°C, suggesting that low temperature affects anther dehiscence by inhibiting PCD of sporophytic tissue-related gene expressions. Yang and Xu (2018) found that in male flowers, concurrently with the binucleate tapetal cell degeneration, the appearance of intercellular spaces and lysis of the stomium region cells lead to anther dehiscence. Conversely, in functionally female flowers, trinucleate tapetum appears with delayed degradation, and the persistent cells with highly vacuolated cytoplasm and stomium region remain intact at maturity. These results indicated that the sporophytic tissues with tapetum abnormalities and stomium integrity were the cause of anther indehiscence. However, Hu and Gao (2022) refuted the previous studies on *C. oleifera* pollen morphology that indicated dual morphologies, defined as ‘dimorphism’ and suggested that the pseudopollen are stomium cells with structure related to their function.

The presence, diversity, and location of these pseudopollens is important in plant identification and taxonomy (Ritchie, 1965). The high degree of uniformity of the camellioid flowers results in reduced use of their floral characters in taxonomy at the tribe and genus levels. For example, Sealy (1958) and Keng (1962) only used fruit and seed characteristics in their keys to tribes, subtribes, and genera of Camellioideae. However, Tsou (1998) observed that anthers in genera traditionally included in Camellioideae all possessed pseudopollen, which was differentiated from connective tissue and had different wall ornamentation in three subgroups. This observation supports the concept that pseudopollen can be used as a taxonomic feature of a subfamily. Concurrently, this has been described as the most important element used for classification in the floral features of the Camellioideae. Pseudopollen with parallel unbranched ridge lines of subgroup I (*Camellia*, *Polyspora*, and *Pyrenaria*), are evolutionarily ancient. In contrast, pseudopollen with pantoporate globe ornamentation of subgroup III (*Franklinia* and *Schima*), such as *Schima superba*, are evolutionarily more recent with more derived states (Tsou, 1996). However, not all members of these genera can produce pseudopollen. For example, pseudopollen has not been identified in *Camellia japonica* or *C. sinensis* (Zhang et al., 2017). Thus, whether pseudopollen micro-characteristics can be used to distinguish them from other species remains unknown.

In this study, we explored the structural characteristics of normal pollen and pseudopollen of *C. oleifera.* To achieve this goal, we compared the morphological and histochemical differences between normal pollen and pseudopollen. We observed and recorded the proportion of the number and morphology of normal pollen and pseudopollens in seven species (*C. hainanica, C. meiocarpa, C. brevistyla, C. confuse, C. hiemalis, C. kissii* and *C. obtusifolia*). Based on the results obtained, we clarified the origin of the pseudopollen and determined differences in the pollen structure and location in the anther of *Camellia* species.

## 2 Materials and methods

### 2.1 Plant material and pollen collection

*C. oleifera* flower buds and anthers were collected from interspecific hybrid YH-3, which has been derived from the F1 generation of interspecific hybrids between *C. yuhsienensis* ‘Hu’(P1) × *C. oleifera ‘*Huashuo’ (P2), in the *C. oleifera* nursery of Central South University of Forestry and Technology, Tianxin District, Changsha City, in eastern Hunan Province (28° 8′ 14′′ N, 112° 59′ 08′′ E). Flower buds were collected once every week from 2 September 2020 to 4 December 2021, which is the period when *C. oleifera* flower buds develop and bloom. All collected flower bud samples were stored in Carnoy’s fixative solution (CF; acetic acid:ethyl alcohol, 3:1, v/v). The anthers were collected in November 2020, which is the period when *C. oleifera* reaches its full flowering stage. In addition, abnormal anthers of *C. oleifera* were collected from Changsha City, Tianxin District, and the flower buds and anthers of *C. hainanica, C. meiocarpa, C. brevistyla, C. confuse, C. hiemalis, C. kissii*, and *C. obtusifolia* from Jinhua City (29°05′32.94″N, 119°38′41.25″E). The anthers were spread over paper sheets in a ‘pollen room’ (28°C, 40% RH, 4000 lx) for one night to release the pollen. Pollen was collected in dry covered 1.5-mL centrifuge tubes and then temporarily stored at 4°C (Luo et al., 2020).

### 2.2 Pollen and pseudopollen morphology

To compare the morphological characteristics of pollen and pseudopollen, we photographed and observed the collected anthers and pollen under a zoom stereoscope microscope (Olympus SZX16, Japan). We recorded the distribution position and color of normal pollen and pseudopollen on the anthers. Then, the dried pollen was plated with gold-palladium under an accelerated voltage of 10 kV, and was observed using a scanning electron microscope (SEM; JEOL SEM-6380LV, Japan). The pollen of *C. oleifera, C. hainanica, C. meiocarpa, C. brevistyla, C. confuse, C. hiemalis, C. kissii* and *C. obtusifolia* were photographed at different magnification (× 500, × 1000, × 2000, × 5000). The characteristics of the wall ornamentation and the aperture of pollen and pseudopollen were recorded. The polar axis length (length of the imaginary line connecting the near and far polar centers of pollen or pseudopollen, P), equatorial axis length (length of the line intersecting at right angles to the polar axis, E), equatorial surface area (area of the pollen shape observed at the equator, s), perimeter (L), mesh area (area of a mesh on the pollen), septum width (length of the septum between two adjacent meshes on the pollen), mesh density (number of meshes per μm^2^ on the pollen), rib interval (distance between two adjacent ribs on the surface of pseudopollen), and rib density (number of ribs per μm^2^ on the pseudopollen) of pollen and pseudopollen were measured using the image processing software Image J 1.6 (National Institutes of Health, Bethesda, USA). Eccentricity (E) was also calculated.

### 2.3 Pseudopollen development

To explore the development processes of the pseudopollen, we placed freshly collected flower buds in CF overnight and then preserved them in 75% ethyl alcohol. The preserved flower bud materials were dissected carefully under a dissecting microscope, then rendered dehydrated and transparent through a graded ethanol series and xylene, infiltrated with liquid paraffin, and embedded in paraplast. The paraplast blocks were sectioned at 10-µm thickness using a manual rotary microtome (Leica RM-2235, China) and stained with glycogen periodic acid–Schiff (PAS) staining solution (Shanghai Yuanye Biological Technology Co., Ltd.). Tissues were then examined microscopically using an inverted microscope (Leica S/M432299, Japan) under green fluorescence and white light (Davies, 2000).

### 2.4 Histochemistry

To confirm the nutrient content in pseudopollen, we conducted histochemical tests for three crucial elements: protein, lipids, and starch. We collected the YH-3 pollen and then prepared aqueous suspensions of pollen for substance determination. A blue reaction product indicated the presence of protein with Coomassie Brilliant Blue. A brown reaction product indicated the presence of starch with iodine–potassium iodide (I-KI). An orange reaction product indicated the presence of lipids with a saturated solution of Sudan III in 70% (v/v) ethanol (Davies et al., 2002).

### 2.5 Transmission electron microscopy

The normal and pseudopollen were fixed in 2.5% glutaraldehyde (pH 7.4) for 2 h. They were washed three times with 0.1 M phosphate buffer (pH 7.2) and fixed in 1% osmic acid at 4°C for 2 h. Next, the samples were dehydrated in a graded series of ethanol. The samples were embedded in Epon-Araldite resin for penetration and placed in a mould for polymerization. A semi-thin section was used for positioning, and an ultrathin section was used for microstructure analysis. After counterstaining the sections with 3% uranyl acetate and 2.7% lead citrate, they were observed under an transmission electron microscope (HT7800, Hitachi, Tokyo, Japan).

### 2.6 Data analysis

#### 2.6.1 One-way analyses

The main morphological characteristics of the pollen grain and pseudopollen grain were analyzed using Tukey-test and one-way analyses of variance. Statistical significance was set at *P* < 0.05. All data analyses were performed using the SPSS 26 version (SPSS Inc., Chicago, USA).

#### 2.6.2 Correlation analysis

The correlation of the micro-characteristics of normal pollen and pseudopollen with species was analyzed by calculating Spearman’s correlation coefficient and using a response ratio of 4.04 (Xu et al., 2021a). The factors that were most strongly related to species were identified.

#### 2.6.3 Cluster analysis

Species were clustered to determine their classification based on micro-characteristics of normal pollen and pseudopollen. The species groups were classified using SPSS (version 26) and the inter-group connection method, and the distance between the species was set as the Euclidean distance (Qian et al., 2020).

## 3 Results

### 3.1 Comparison between pollen and pseudopollen

**Figure 1:**
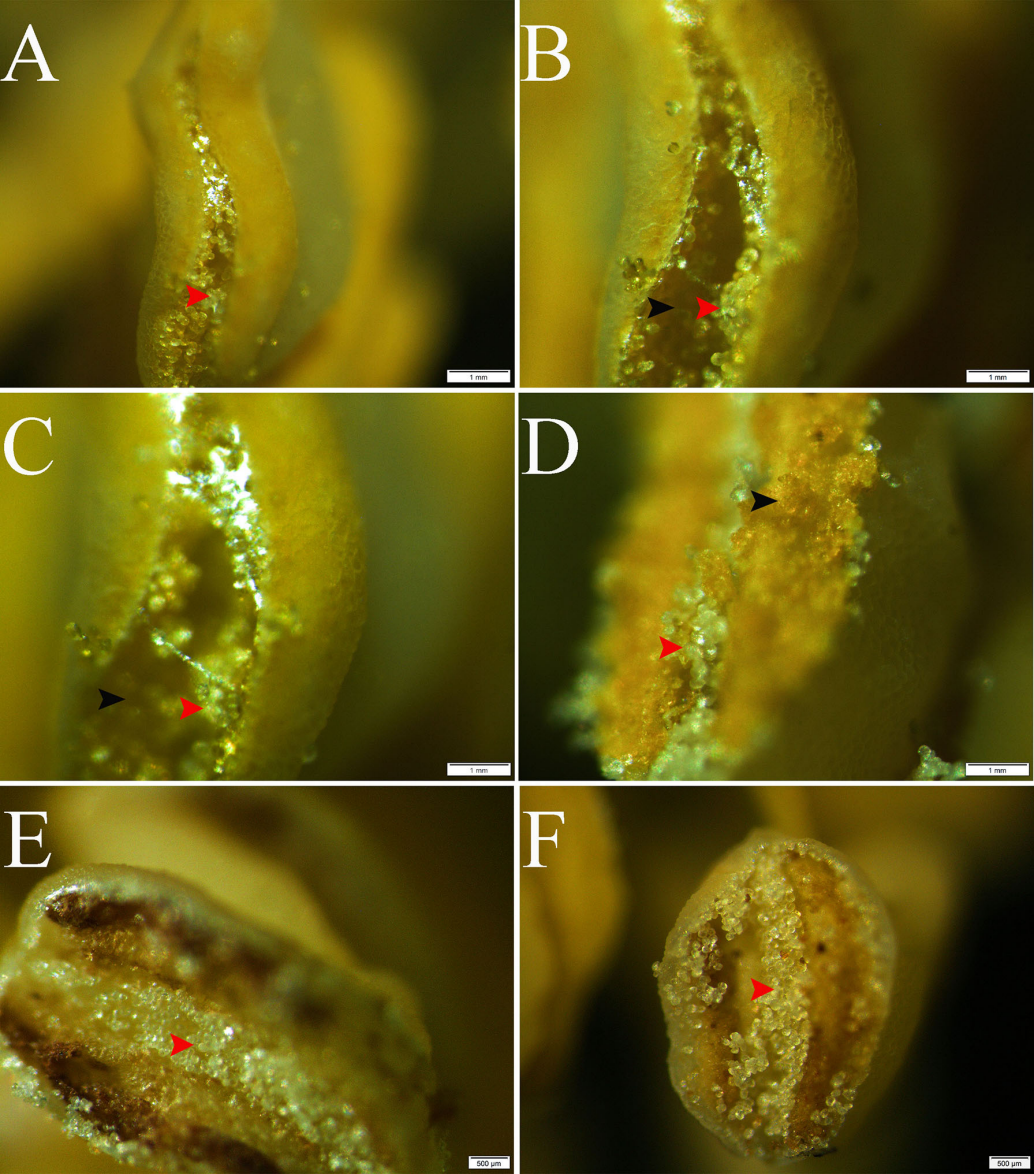
Release and distribution of normal and pseudopollen in *Camellia oleifera* normal anthers and abnormal anthers. **(A–D)** Normal anthers. **(E, F)** Anthers that do not normally produce fertile pollen. Bar = 1 mm.

The stereo microscope images showed a regular distribution with alternating normal pollen and pseudopollen on each side of the anther of *C. oleifera*. The normal pollen was bright yellow and distributed on both sides of the connective tissue, whereas the pseudopollen was white and transparent, distributed in the connective tissue (**Figures 1A–D**). In addition, observation of abnormal anthers revealed evident pseudopollen grains in the connective position, regardless of whether the pollen developed normally, as long as the anthers cracked normally (**Figures 1E, F**).

**Figure 2:**
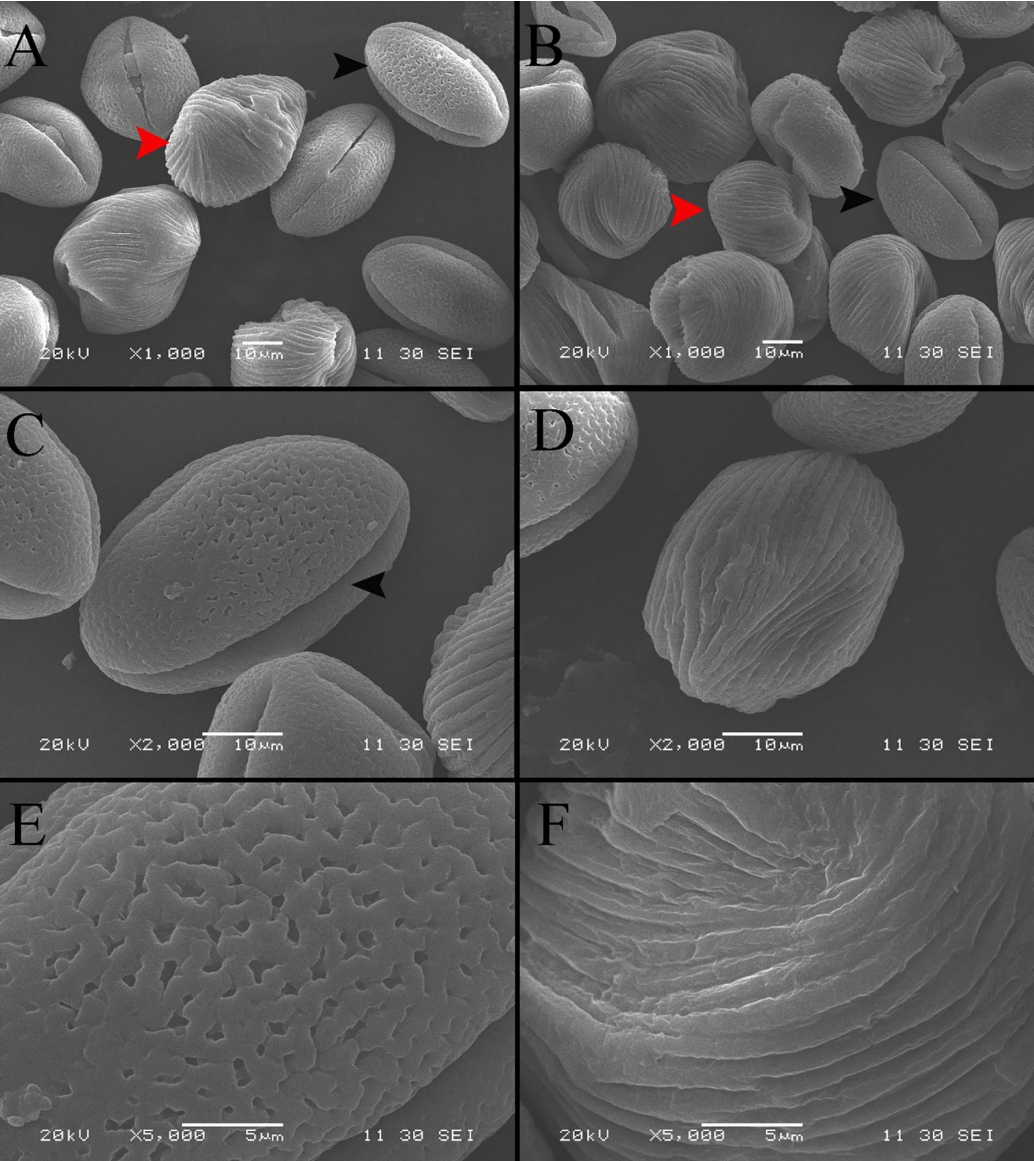
Comparison of morphological characteristics of pollen and pseudopollen of *Camellia oleifera* under SEM. **(A, B)** Morphological differences between normal pollen grains and pseudopollen grains. The black arrow indicates normal pollen; the red arrow indicates pseudopollen. 2 **(C)** Complete morphology of normal pollen grains. The black arrow indicates a pollen aperture **(D)** Complete morphology of pseudopollen grains **(E)** Grid ornamentation of the exine of normal pollen **(F)** Parallel ribs in the wall of pseudopollen grains.

The SEM images display the differences in the morphology and ornamentation of normal pollen and pseudopollen (**Figures 2A, B**). The equatorial surface area and axis length of pseudopollen grains were similar to those of normal pollen grains. The equatorial planes of normal pollen and pseudopollen were flattened, with eccentricities > 0.6. However, the eccentricity of the pseudopollen was significantly lower than that of the normal pollen, indicating that pseudopollen is more round than normal pollen (**Table 1**). In contrast to the regularity of normal pollen, the surface of the pseudopollen was often irregular, with depressions or uplift. Normal pollen was three colporate. whereas pseudopollen did not (**Figures 2C, D**). Meanwhile, the ornamentation of exine of normal pollen was reticulate, while the pseudopollen had almost wall depositions in a striate pattern (**Figures 2E, F**).

**Table 1:** Normal and pseudopollen morphology of different species of *Camellia*.

Categories	Species	Area (μm2)			Polar axis length(μm)	Equatorial axis length(μm)	Perimeter(μm)	Eccentricity	Mesh area(μm2)	Septum width(μm2)	Mesh density(μm-2)	Rib interval(μm)	Rib density(μm-2)	Ornamentation	Aperture
		Average	Maximum	Minimum											
Normal pollen	*Camellia oleifera*	1022.665 ± 145.180 b	1282.990	480.843	30.670 ± 3.849 a	43.013 ± 5.674 b	119.632 ± 11.043 a	0.672 ± 0.139 c	0.313 ± 0.187 b	0.735 ± 0.213 bc	0.778 ± 0.184 c	/	/	reticulate	+
	*Camellia hainanica*	1292.024 ± 277.455 a	1742.686	821.657	30.299 ± 4.295 a	53.544 ± 5.160 a	95.240 ± 18.719 c	0.823 ± 0.035 a	0.233 ± 0.061 c	0.178 ± 0.048 g	0.402 ± 0.102 d	/	/	+
	*Camellia meiocarpa*	677.153 ± 80.310 d	837.250	528.122	26.917 ± 2.264 b	30.109 ± 2.161 e	86.379 ± 10.877 d	0.411 ± 0.157 e	0.121 ± 0.016 d	0.655 ± 0.276 cd	1.768 ± 0.634 a	/	/	+
	*Camellia brevistyla*	748.669 ± 142.878 cd	976.724	634.233	24.441 ± 5.645 c	39.265 ± 2.500 c	105.191 ± 6.225 b	0.743 ± 0.158 b	0.135 ± 0.037 d	0.584 ± 0.102 de	0.868 ± 0.174 c	/	/	+
	*Camellia confusa*	799.036 ± 292.859 c	1749.754	494.696	27.188 ± 6.400 b	36.247 ± 5.579 d	104.007 ± 15.809 b	0.637 ± 0.135 c	0.088 ± 0.042 d	0.751 ± 0.240 b	0.913 ± 0.428 b	/	/	+
	*Camellia hiemalis*	723.854 ± 97.020 d	804.864	598.181	24.399 ± 0.994 c	36.115 ± 3.192 d	100.388 ± 7.343 bc	0.733 ± 0.032 b	0.213 ± 0.048 c	0.955 ± 0.146 a	0.698 ± 0.123 c	/	/	+
	*Camellia kissii*	569.701 ± 52.518 e	681.355	488.381	24.388 ± 1.492 c	29.320 ± 1.770 e	87.178 ± 4.015 d	0.542 ± 0.116 d	0.204 ± 0.110 c	0.505 ± 0.215 ef	0.908 ± 0.210 c	/	/	+
	*Camellia obtusifolia*	462.984 ± 61.928 f	588.428	354.545	18.515 ± 2.041 d	29.018 ± 3.535 e	82.115 ± 7.085 d	0.765 ± 0.084 ab	1.535 ± 0.283 a	0.485 ± 0.135 f	0.877 ± 0.262 c	/	/	+
Pseudopollen	*Camellia oleifera*	1109.365 ± 251.918 b	1802.974	683.776	30.508 ± 5.221 a	45.576 ± 5.605 b	125.959 ± 13.283 a	0.741 ± 0.063 a	/	/	/	1.647 ± 0.274 a	0.096 ± 0.034 d	composed of many unbranched rib	–
	*Camellia hainanica*	1223.058 ± 202.022 a	1584.556	881.291	29.083 ± 6.179 a	49.437 ± 7.466 a	72.600 ± 5.313 e	0.786 ± 0.102 a	/	/	/	1.315 ± 0.466 b	0.095 ± 0.026 d	–
	*Camellia meiocarpa*	856.086 ± 140.742 c	1147.493	539.247	22.519 ± 3.838 c	37.537 ± 5.399 c	74.692 ± 8.514 e	0.787 ± 0.094 a	/	/	/	1.383 ± 0.342 b	0.135 ± 0.073 d	–
	*Camellia brevistyla*	653.629 ± 94.025 d	846.000	532.997	26.272 ± 3.038 b	31.672 ± 1.071 de	92.836 ± 1.986 c	0.604 ± 0.092 d	/	/	/	1.115 ± 0.245 c	0.038 ± 0.014 e	–
	*Camellia confusa*	572.290 ± 115.305 e	730.054	395.130	22.012 ± 3.828 c	33.914 ± 4.489 d	91.362 ± 9.512 c	0.746 ± 0.097 ab	/	/	/	1.028 ± 0.338 c	0.238 ± 0.129 b	–
	*Camellia hiemalis*	815.653 ± 104.587 c	1024.052	608.601	26.789 ± 4.265 b	39.928 ± 1.802 c	105.815 ± 1.734 b	0.749 ± 0.068 a	/	/	/	1.295 ± 0.297 b	0.201 ± 0.058 c	–
	*Camellia kissii*	513.104 ± 170.848 e	834.098	244.606	21.071 ± 4.225 c	27.319 ± 5.373 f	81.310 ± 15.241 d	0.613 ± 0.164 cd	/	/	/	0.757 ± 0.454 d	0.390 ± 0.217 a	–
	*Camellia obtusifolia*	509.787 ± 115.951 e	769.114	288.340	20.669 ± 4.214 c	29.353 ± 4.351 ef	88.866 ± 9.936 c	0.671 ± 0.161 bc	/	/	/	1.338 ± 0.390 b	0.080 ± 0.041 de	–

Within columns, different letters indicate significant differences at P <0.05.

“+”, aperture presence; “-”, aperture absence.

**Figure 3:**
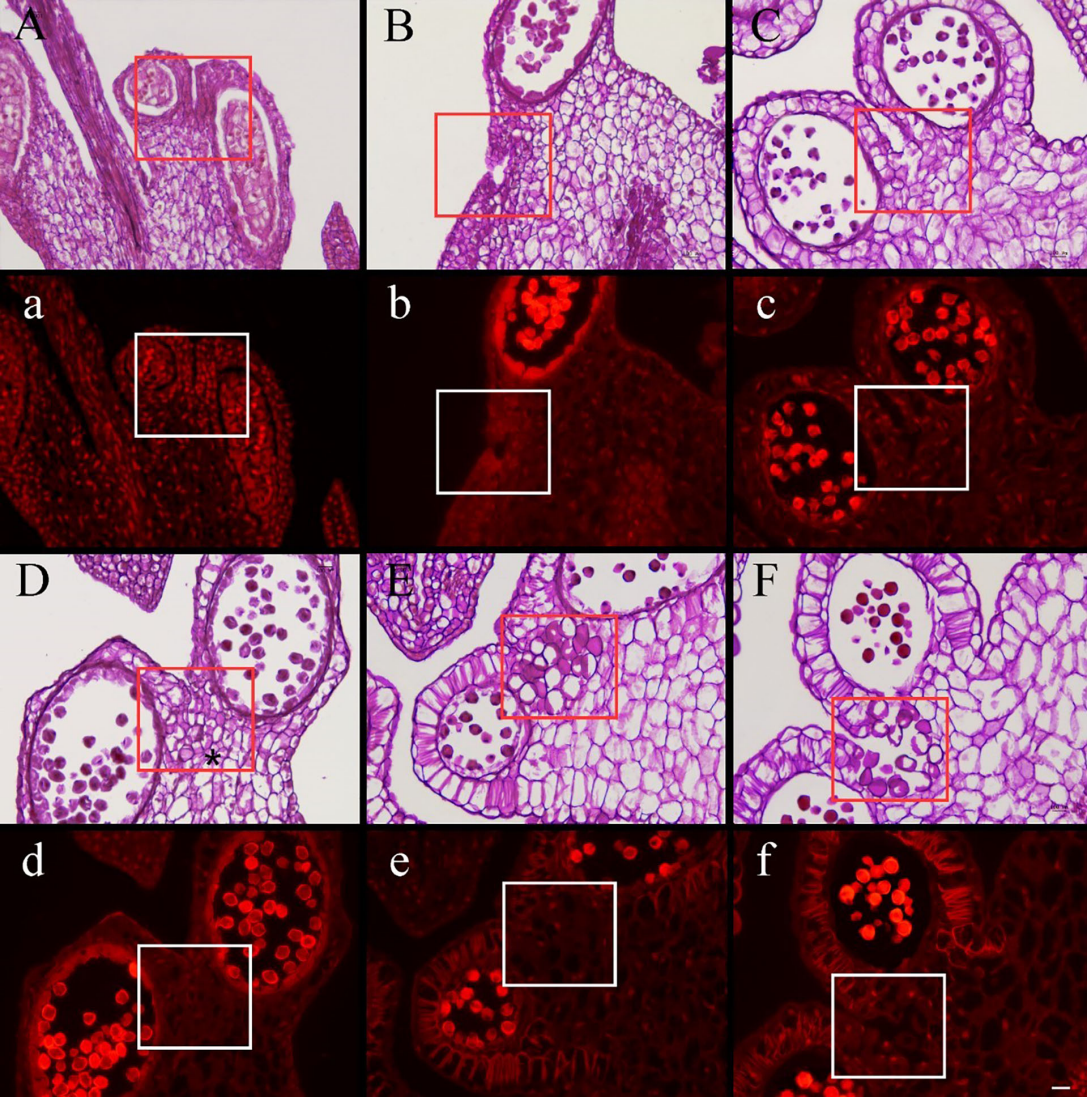
Cross section stained with periodic acid–Schiff (PAS) of *Camellia oleifera* anthers during the development stages of pseudopollen under light microscope **(A–F)** and fluorescence microscope **(a–f)**. **(A, B)** The cells of connective are undifferentiated. **(a, b)** Polysaccharides show a red reaction under the fluorescence of connective cells. **(C)** The parenchyma cells gradually begin to form free ellipsoid cells. **(D)** Adjacent cells are pressed to form a boundary (*). **(E)** The pseudopollen grains are fully expanded. **(F)** The pseudopollen grains separate from each other. **(c–f)** In the process of pseudopollen development, the pseudopollen grains are almost completely black under fluorescence. The red and white rectangle represents the site where the pseudopollen is produced. Bar = 100 μm.

Research on the development of pseudopollen revealed that, at the early sporangial developmental stage, the mesophyll cells of the connective tissue are enlarged; however, the cells between the two pollen sacs, which would eventually differentiate into septa, remain dense, small, and undifferentiated (**Figures 3A, B**). Simultaneously, polysaccharides are clearly visible in the connective tissue under fluorescence (**Figures 3A, B**). Subsequently, the connective tissue cells commenced complex morphological changes; the polysaccharide content of the cells that would transform into pseudopollen grains appeared to decrease gradually over time (**Figures 3C–F**).

**Figure 4:**
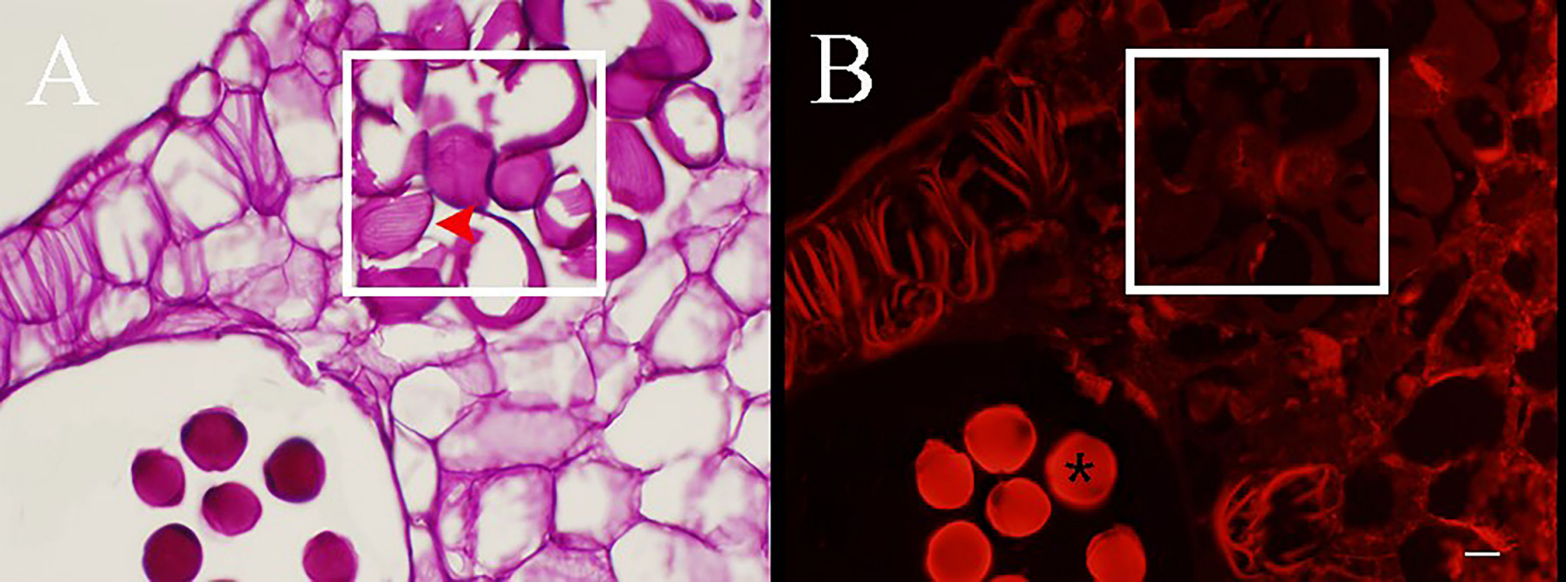
PAS staining sections of anthers during the development of pseudopollen of *Camellia oleifera*. **(A)** Obvious parallel ribs can be observed on the wall of pesudopollen grains (red arrow). **(B)** Under fluorescence, the pseudopollen grains are black whereas the normal pollen grains are bright red (*). The white rectangle represents pseudopollen. Bar = 50 μm.

Concomitant with the development of pollen mother cells within the sac, the parenchyma cells of the connective cells initially expanded and gradually transformed into dissociative oval to round cells through the decomposition of their intermediate lamellae and the creation of intercellular spaces (**Figure 3C**). Meanwhile, the wall of the connective cells underwent secondary thickening, several unbranched ribs began to form, and the pseudopollen grains contained almost no polysaccharides (**Figures 4A, B**). Along with the pseudopollen expansion, the adjacent connective cells were gradually, horizontally compressed, resulting in a well-defined boundary distinguishing the pseudopollen grains and connective cells (**Figure 3D**). During late differentiation, pseudopollens were separated, although they appeared as a whole and were compacted together at the location of the septum (**Figure 3E**). During the late uninucleate to binucleate stages of microsporogenesis, the wall between the pollen sacs stomia broke at the anthesis and the pseudopollen grains separated to form a relatively loose structure, showing a tendency to enter the pollen sacs; however, most of the pseudopollen grains were still distributed at the middle of the pollen sacs. Only some pseudopollens, distributed at the circum of the pollen sacs, entered the pollen sacs (**Figure 3F**).

**Figure 5:**
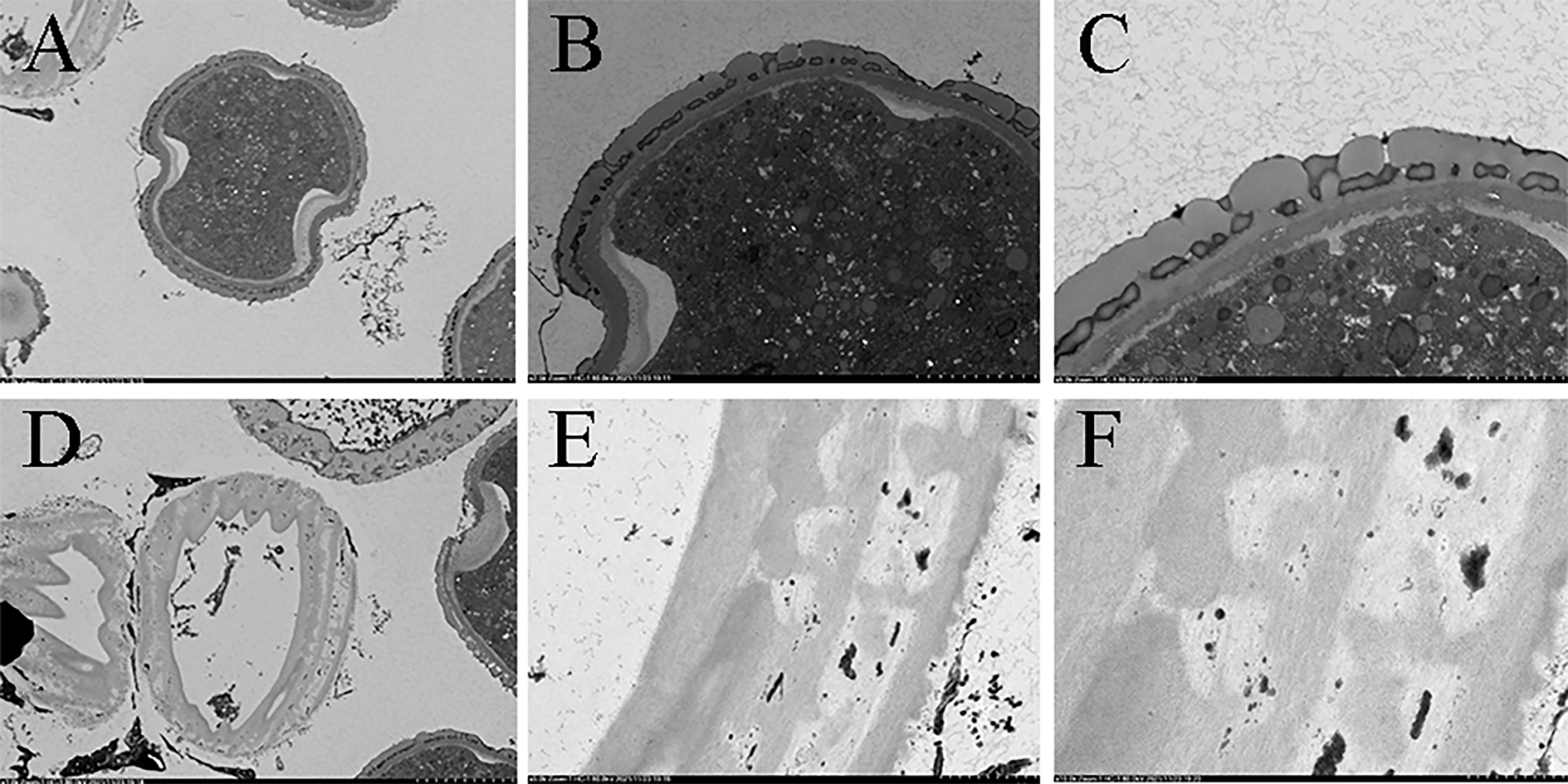
Comparison of internal structure and the wall of pollen and pseudopollen of *Camellia oleifera* under TEM. **(A–C)** Normal pollen. **(D–F)** Pseudopollen.

In terms of cytology, the results of transmission electron microscopy recorded significant differences in the internal structures and walls between normal pollen and pseudopollen. The internal vacuolization of pseudopollen is severe—wherein the organelles are severely degraded or even decomposed—and it is difficult to identify the complete organelle structure, and the pseudopollens finally shrink into protein clusters. The outer wall of pseudopollen shows uneven secondary thickening, thus forming rib ornamentation. The outer walls of secondary growth are smooth and the walls of pseudopollen grains are ribbed ridge, and the cell wall is concave during its development (**Figures 5D−F**). In contrast, the outer wall of normal pollen has a discontinuous and perforated covering layer and secretes pollenkitte, an oil substance. Unlike vacuolated pseudopollen, the interior of normal pollen is full of starch granules, with sperm and vegetative cells (**Figures 5A−C**).

**Figure 6:**
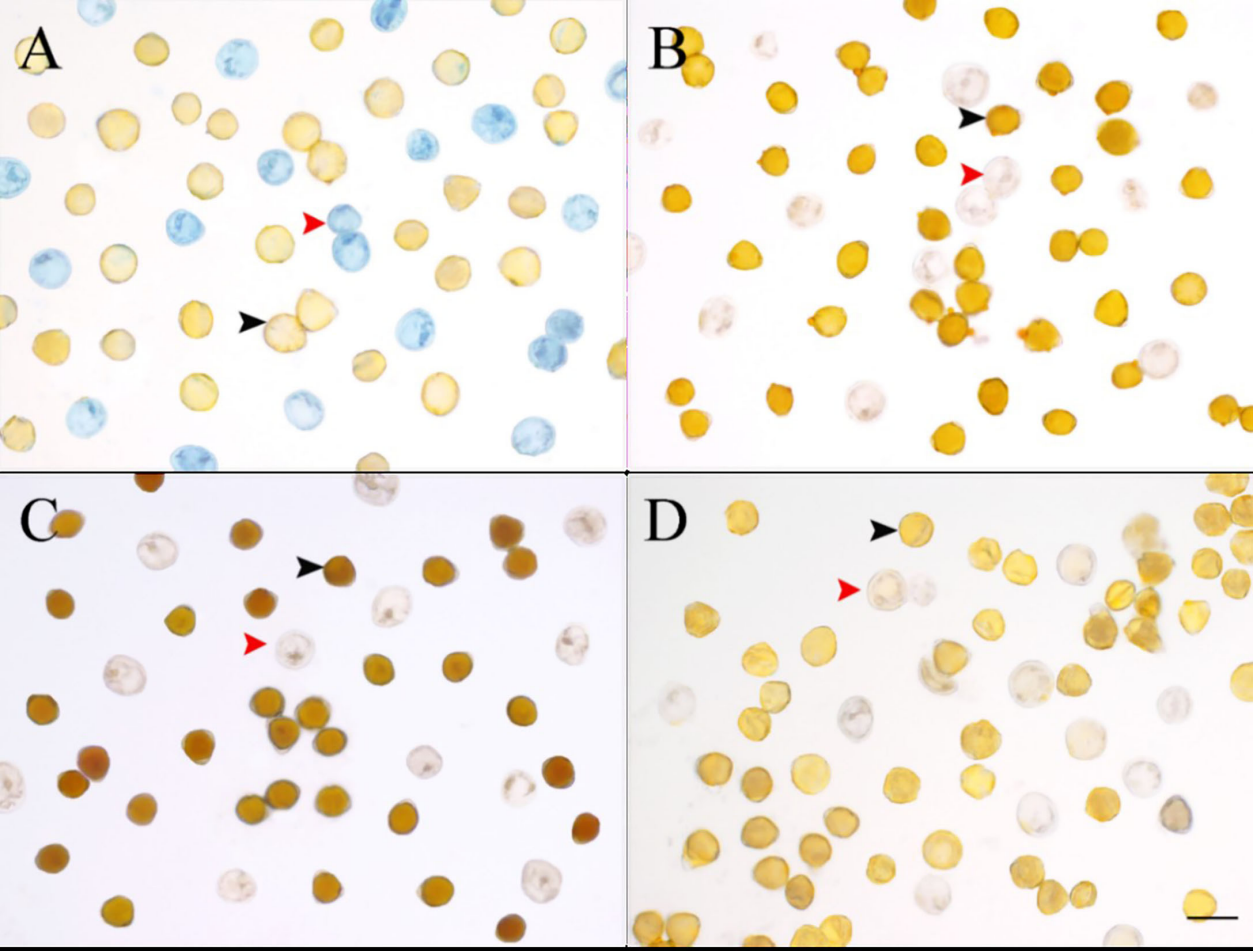
Comparison of four stains for normal pollen and pseudopollen of *Camellia oleifera*. **(A)** Coomassie Brilliant Blue. **(B)** Sudan III. **(C)** I-KI. **(D)** Control group. The black arrow indicates normal pollen; the red arrow indicates pseudopollen. Bar = 50 μm.

The histochemical tests showed that *C. oleifera* pseudopollen was rich in proteins (**Figures 6A, D**) but had almost no lipid content (**Figures 6B, D**) and contained a small amount of starch (**Figures 6C, D**). However, the normal pollen was mainly composed of starch (**Figures 6A, D**) and had almost no protein (**Figures 6C, D**), and only part of the pollen aperture was stained by Sudan III (**Figures 6B, D**).

### 3.2 Normal and pseudopollen morphology of other species

**Figure 7:**
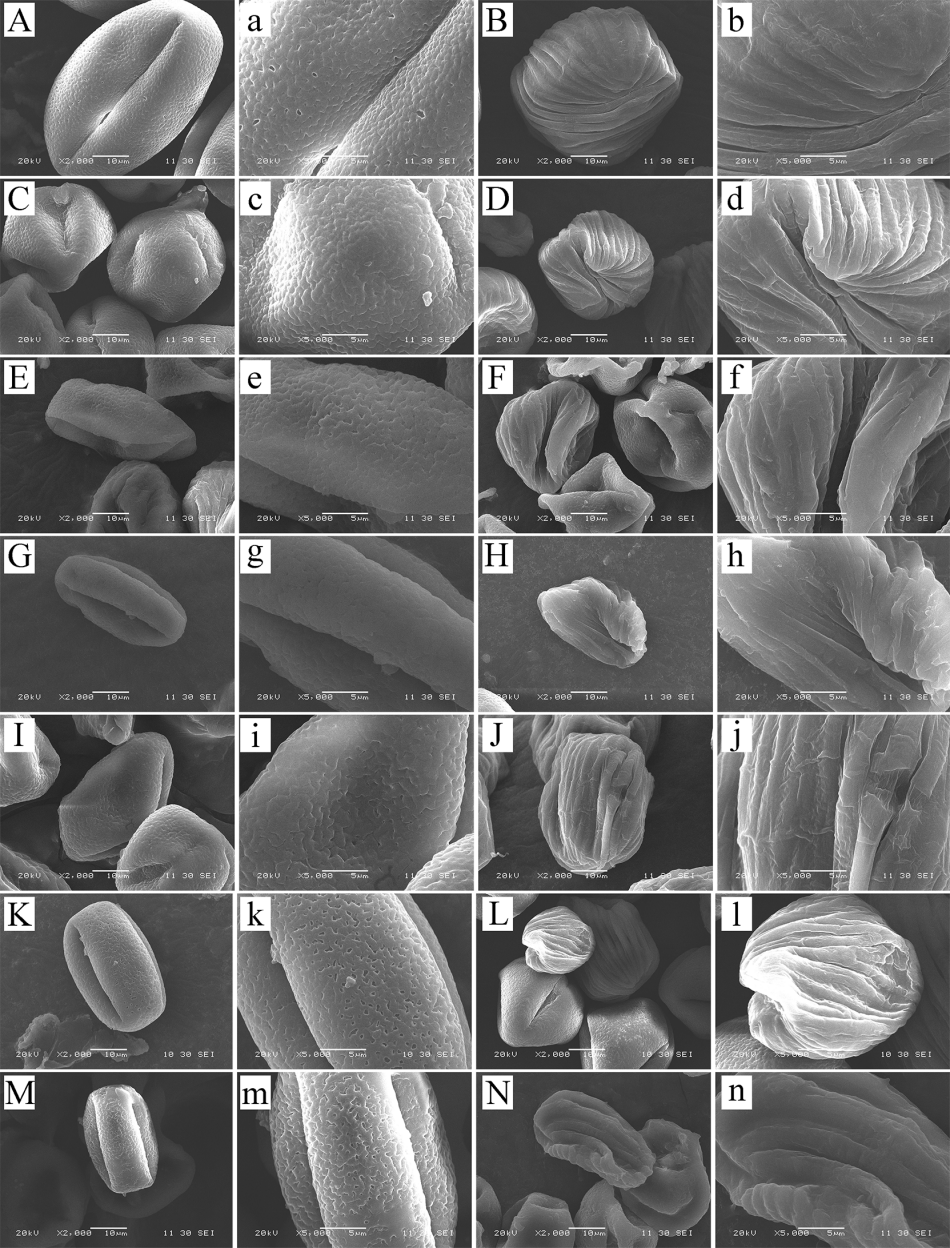
Normal pollen and pseudopollen of different species of *Camellia* under scanning electron microscopy. **(A, B)** *Camellia hainanica.* **(C, D)** *Camellia meiocarpa.* **(E, F)** *Camellia brevistyla.* **(G, H)** *Camellia confusa.* **(I, J)** *Camellia hiemalis.* **(K, L)** *Camellia kissii.* **(M, N)** *Camellia obtusifolia*. The two columns of pictures on the left are normal pollen, and the right are pseudopollen.

We studied eight species in total: *C. oleifera*, *C. hainanica* (**Figures 7A, D**), *C. meiocarpa* (**Figures 7C, D**), *C. brevistyla* (**Figures 7E, F**), *C. confuse *(**Figures 7G, H**), *C. hiemalis *(**Figures 7I, J**), *C. kissii *(**Figures 7K, L**), and *C. obtusifolia *(**Figures 7M, N**). Measurements of the characteristics of the wall ornamentation and aperture demonstrated significant differences in polar axis length (P), equatorial axis length (E), equatorial surface area (S), and perimeter (L) between pollen and pseudopollen. Significant differences in the ornamentation of normal pollen and pseudopollen in different species were observed. The pollen grain surfaces of all eight species were perforate to reticulate, while those of pseudopollen were rib shaped. Generally, the larger the normal pollen, the larger the pseudopollen (**Table 1**)*. Camellia hainanica* had the largest normal pollen and pseudopollen. In the normal pollen of *C. oleifera*, the mesh was larger, transverse septum was wider, and mesh density was smaller, while in that of *C. meiocarpa* and *C. confuse*, the mesh was smaller and unevenly distributed. The mesh of *C. hainanica* was unclear and the pollen surface was the smoothest. The rib of the pseudopollen of *C. oleifera* was the widest and deepest (**Figure 7**).

**Figure 8:**
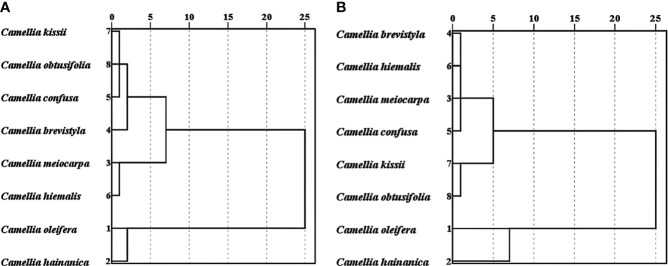
Clustering of normal pollen and pseudopollen micro-characteristics related to species. **(A)** Normal pollen. **(B)** Pseudopollen.

The correlation analysis determined that not every micro-characteristic index showed significant correlation with species. In normal pollen, eccentricity and mesh area were not significantly correlated with species; while in pseudopollen, perimeter was not significantly correlated with species (**Table 2**). The cluster analysis, using indices that exhibited significant correlation with species, demonstrated that the normal pollen (**Figure 8A**) and pseudopollen (**Figure 8B**) micro-characteristic clustering were similar and both grouped *C. oleifera* and *C. hainanica* together. However, slight differences in the clustering results of the other six species were observed (**Figure 8**).

**Table 2:** Correlation between micro-characteristics of pollens (normal pollen and pseudopollen) and species.

Categories	Area	Polar axis length	Equatorial axis length	Perimeter	Eccentricity	Mesh area	Septum width	Mesh density	Rib interval	Rib density
Normal pollen	-0.75**	-0.46**	-0.62**	-0.63**	-0.03	0.11	-0.39**	0.13*	/	/
Pseudopollen	-0.76**	-0.12	-0.71**	-0.49**	-0.25**	/	/	/	-0.31**	0.29**

*T-test for all variables, P < 0.05. **T-test for all variables, P < 0.01.

## 4 Discussion

Although pseudopollen in Theaceae has been previously reported, little is known about its specific characteristics. In this study, we determined the morphological characteristics and occurrence of pseudopollen in *C. oleifera*. We also demonstrated that protein is the main component of *C. oleifera* pseudopollen. Our study is the first to investigate these aspects of *C. oleifera* pseudopollen. In addition, we compared the proportion and morphological characteristics of normal pollen and pseudopollen of seven species (*C. oleifera, C. hainanica, C. meiocarpa, C. brevistyla, C. confuse, C. hiemalis, C. kissii* and *C. obtusifolia*), and found that it may be a basis for the classification of *Camellia*.

### 4.1 Occurrence of pseudopollen in *C. oleifera*

The pseudopollen of *C. oleifera* was derived from parenchyma cells of the connective tissue, which is consistent with other species in the genus but different from that of Orchidaceae, which is formed by fragmentation of multicellular moniliform trichomes cells (Davies and Turner, 2004; Jersakova and Johnson, 2006; Davies et al., 2013). Cell differentiation of the parenchyma of the connective tissue occurs when the pollen mother cell enters the tetrad stage. This process involves the decomposition of the middle lamellae, expansion of connective cells, secondary thickening of the cell wall, cracking of the tapetum, and evacuation of pseudopollen grains.

The pseudopollen distribution pattern in *C. oleifera* differs from *Gordonia dassanayakei* and *G. ceylanica* as it was distributed in the middle of the two pollen sacs rather than entering the pollen sacs; however, minimal marginal pseudopollen was mixed into the anther sac (Iqbal and Wijesekara, 2002). On one side of the anther, a regular distribution with alternating normal pollen and pseudopollen on each side of the anther was observed. Pseudopollen did not enter the pollen sac at a later stage; therefore, pseudopollen and normal pollen were concentrated in their respective positions, which is conducive to the location and acquisition of pollinators (Davies et al., 2000). This distribution pattern suggests that the cracking of the *C. oleifera* anther may be controlled by pseudopollen rather than by producing fracture tissue, as in other plants, such as watermelon (Yang and Xu, 2018; Lyu et al., 2019; Hu and Gao, 2022). Hu and Gao (2022) determined that the pseudopollen of *Camellia oleifera* realized anther cracking through dehydration to ensure this occurred on sunny days. The stomium cells in anthers produce striated cell walls after maturation, the nucleus of the inner stomium cells are located in the direction of the connective, and the septum is formed. The septum is outside the stomium cells and the dehydration of the stomium and epidermal cells pull the septum, causing the connective cells and pseudopollen to separate. During the flowering stage, the pollen sacs are broken and the pseudopollens are released into the pollen sac, where they mix with the pollen grains. The inner part of pseudopollen observed under TEM was almost hollow. This is possibly because the framework of their cell walls is composed of unbranched edges. On this basis, we hypothesize that the existence of pseudopollen is a pollen presentation strategy for *C. oleifera* to protect pollen from waste (Xu et al., 2021b). Additionally, with the development and maturity of pseudopollen, intracellular polysaccharide content gradually decreased. Intracellular polysaccharides can be stained pink/purple by PAS, and fluorescence microscopy can clearly distinguish between non-polysaccharides with strong fluorescence reactions and insoluble polysaccharides without fluorescence (de Oliveira et al., 2015; Song et al., 2015; Chawla et al., 2017). In our study, the early maturing cells were rich in polysaccharides. Moreover, the staining site was highly consistent with the reflected fluorescence site, indicating that the cells contained many non-polysaccharides, but starch was not excluded. With the progressing maturity of pseudopollen, the color/fluorescence gradually became lighter and ultimately almost colorless, that is, possessing almost no polysaccharides. This may indicate that pseudopollen is easier to dehydrate than normal pollen, which further infers that pseudopollen can regulate anther dehiscence through dehydration (Pacini, 2000; Vesprini et al., 2002).

### 4.2 Pollination strategy combined with both reward and deception

The resemblance of pseudopollen to normal pollen makes it capable of attracting pollinators solely by mimicry, as proposed by Vogel (1978). The pseudopollen grains produced by Theaceae species morphologically resemble monad pollen grains. It did not differ significantly from normal pollen in the cross-sectional area and was ellipsoidal. Furthermore, it is transported along with pollen grains by pollinators (Yuan et al., 2022). The pseudopollen of *C. oleifera* had almost identical morphology to that of real pollen; therefore, we speculated that the pseudopollen might convey deception signals to insects by simulating the morphology of normal pollen. Pollen fluorescence, which attracts pollinators by highlighting pollen as food, has been proposed as one of the visual cues for insects (Mori et al., 2018). The pseudopollen produced by *C. oleifera* was similar in shape to normal pollens and was produced by anthers like pollen, but there were differences in color between them. We believe that the “false” pollen of *C. oleifera* imitates the normal pollen by visual signals such as ultraviolet light (UV)-absorbing visual patterns, transmitting this visual signal to the receivers of bees and flies (Sanguinetti et al., 2012; Papiorek et al., 2016). In addition, the distribution pattern of pseudopollen also enhanced the visual stimulation to insects (Davies et al., 2006). The surface of the pseudopollen is covered with ribs, which enables the pseudopollen to capture more light, thus enhancing the visual stimulation to insects (Kay et al., 1981). However, an individual pollen grain is too small to present a visual signal in itself (Lunau, 2000). Therefore, the pseudopollen is centrally distributed to ensure the successful expression of this visual signal.

We found that pseudopollen plays a significant role in attracting pollinators. Instead of leaving the insects ‘empty-handed’ after being lured by deception, pseudopollen rewards visiting insects in the form of edible materials (Jiang et al., 2020). When insects visit flowers, they need to consume pollen to extract protein, which provides them with nutrients (Gilbert, 1981; Gittings et al., 2006). However, histochemical tests showed that *C. oleifera* pollen contained almost no protein, whereas its pseudopollen had significant amounts of protein. Furthermore, in our previous study, we found that the main visiting insects of *C. oleifera* were wasps and flies; additionally, pseudopollen was found in their intestines and pollen baskets, suggesting that they all collected pseudopollen (Singer and Koehler, 2004; Pansarin et al., 2006; Pansarin and Maciel, 2017; Yuan et al., 2022a). Therefore, we believe that pseudopollen in *C. oleifera* may be in the form of edible substances as a reward for visiting insects.

In summary, the white pseudopollen of *C. oleifolia* is not a simple deception or reward, but a pollination strategy combining both (Johnson and Schiestl, 2016; Zheng et al., 2021). Why did *C. oleifera* evolve this pollination strategy? We believe that the pseudopollen of *C. oleifera* may have evolved to promote generalized pollination, resulting in more efficient reproduction (Waser et al., 1996; Alarcón et al., 2008). *C. oleifera* can attract many wasps and flies to pollinate it by means of pseudopollen, which is beneficial for its reproduction in different habitats, especially when pollinators are insufficient. Unlike plants that flower in the spring, the anthesis of *C. oleifera* occurs in the fall with fewer pollinating insects, thus requiring the use of “dual attraction” to attract more insects for pollination to achieve reproductive success.

### 4.3 Classification and circumscription

Pseudopollen can be used as a taxonomic feature of the species (Joanna et al., 2020). Pollen morphology has long been one of the most reliable taxonomic tools, and the aperture and the wall sculpturing are considered the most important characters used to classify pollen grains (Basak et al., 2022). In Theaceae, in addition to normal pollen, pseudopollen is also considered as an important taxonomic basis of subgenus. In fact, pseudopollen can also be used as a taxonomic feature of *the* infrageneric group. In *Camellia*, the pseudopollen of the species *C. oleifera, C. hengchunensis, C. sinensis* (Tsou, 1998), and *C. japonica* (Zhang et al., 2017) have been previously studied. *C. oleifera* and *C. hengchunensis* have pseudopollen decorated with parallel unbranched ridge lines, whereas *C. sinensis* and *C. japonica* do not. Therefore, we speculate that *C. oleifera* and *C. hengchunensis* belong to the same group, while *C. sinensis* and *C. japonica* belong to another group. Vijayan et al. (2009) divided Camellia into eight main branches by analyzing the NRITS sequence: *C. sinensis* and *C. japonica* belong to branch D (Thea clade) and E (species from *Camellia-2* and *Paracamellia-2*), *C. oleifera* and *C. hengchunensis* belong to branch F (species from *Oleifera* and *Tuberculata-2*). They suggested that branches D and E come from the same branch with a parallel evolutionary relationship with group F, which is consistent with our conjecture. Based on the SEM observation and measurements, *Camellia* can be identified to species level according to size and ornamentation of pseudopollen. By comparing the normal and pseudopollen of eight species of *Camellia*, we determined that differences still existed despite possessing the same ornamentation types as pseudopollen (Ahmad et al., 2022). For example, the pseudopollen of *C. hainanica* is larger than *C. oleifera* and the rib interval is wider.

Our results suggest that pseudopollen should be used as a taxonomic feature of interspecific micromorphology. However, we cannot judge whether it is more accurate to use normal pollen as a classification index or pseudopollen because taxonomic feature needs to be linked to molecular phylogenetic analysis, such as linked morphological features and nrITS phylogeny (Yin et al., 2022). However, some of the investigated species, such as *C. hainanica*, have not yet obtained their gene sequences; therefore, more in-depth investigations to explore the taxonomic significance of pseudopollen in *Camellia* are required.

## Data availability statement

The original contributions presented in the study are included in the article/supplementary material. Further inquiries can be directed to the corresponding authors.

## Author contributions

BY, formal analysis, validation, methodology, and writing - original draft. J-KY, writing - original draft. C-GH, picture shooting. J-RL, validation. Y-HL, formal analysis. X-MF, supervision, validation, writing—review, and editing. D-YY, project administration, conceptualization, and resources.

## Funding

This research was supported by the National Key R&D Program of China (2018YFD1000603-1). Natural Science Foundation of Hunan Province (grant no. 2022JJ30997).

## Acknowledgments

We thank Yi-bo Luo of the Institute of Botany, the Chinese Academy of Sciences for his advice on this research.

## Conflict of interest

The authors declare that the research was conducted in the absence of any commercial or financial relationships that could be construed as a potential conflict of interest.

## Publisher’s note

All claims expressed in this article are solely those of the authors and do not necessarily represent those of their affiliated organizations, or those of the publisher, the editors and the reviewers. Any product that may be evaluated in this article, or claim that may be made by its manufacturer, is not guaranteed or endorsed by the publisher.
